# Recent Technical Developments in the Field of Laparoscopic Surgery: A Literature Review

**DOI:** 10.7759/cureus.22246

**Published:** 2022-02-15

**Authors:** Lama Islem Basunbul, Lenah Sulaiman S Alhazmi, Shahad Amro Almughamisi, Najd Muhammed Aljuaid, Hisham Rizk, Rana Moshref

**Affiliations:** 1 Surgery Department, King Abdulaziz University, Jeddah, SAU; 2 Surgery Department, University of Jeddah, Jeddah, SAU

**Keywords:** laparoscopic surgery (ls), advances, abdominal surgeries, robot-assisted laparoscopic surgery (rals), laparoscopic cholecystectomy (lc)

## Abstract

The benefits of laparoscopic surgery (LS) include a speedy recovery, shorter duration of hospital stay, minimal postoperative pain, discomfort and disabilities, and better cosmetic outcomes (less scarring) that help an individual to resume normal daily activities and return to work. A comprehensive literature search on laparoscopic surgeries was conducted using different Internet-based search engines and databases from August 2021 to October 2021. The search was limited to articles published in the English language and those published between years 2005 and 2021. A total of 126 articles were initially identified. Two independent reviewers thoroughly examined the quality and content of the articles. Articles with duplicate data were excluded, and the remaining articles were screened and assessed by the titles and abstracts. After a vigorous assessment, we included data from 49 articles for this review process. Bibliographic management was done using the software “EndNote” (Thomson Reuters, New York, NY, USA).

It was concluded that LS has become the technique of choice for virtually every kind of abdominal surgery, evident by numerous scholarly publications in this field. Level I evidence demonstrating the advantage of LS over open surgery has been reported for numerous operations, including fundoplication for gastroesophageal regurgitation disease, bariatric surgery for weight loss, and cancer resection. Advanced LS has subsequently been expanded to include hepatectomy, pancreatectomy, urology, and gynecology. Patients who are at risk of having elevated abdominal pressure during LS, however, should proceed with care. Recent advances in natural orifice transluminal endoscopic surgery, single-incision laparoscopic surgery, and robot-assisted laparoscopic surgery are promising.

## Introduction and background

The first laparoscopic cholecystectomy was done in 1987 by Philippe Mouret. Since then, laparoscopic surgery (LS) has been considered the gold standard in the treatment of many abdominal disorders such as gynecological problems, cholecystitis, and appendicitis [1]. Some of the benefits of LS include a speedy recovery, shorter duration of hospital stay, minimal postoperative pain, discomfort and disabilities, and better cosmetic outcomes (less scarring) that help the individual to resume normal daily activities and return to work [2,3]. Over the last decades, this surgical procedure has undergone tremendous and exciting advancements and has shown good results with some conditions that were once contraindicated for LS, which include cancer, obesity, abdominal hernia, pregnancy, previous laparotomies, previous abdominal surgeries, and bowel perforation with generalized peritonitis [4].

Minimally invasive surgery (MIS) techniques are rapidly evolving through meticulous scientific approaches, and new methods are constantly being introduced. Despite the benefits these surgeries offer, there are numerous technical limitations encountered by surgeons. Compared to open surgeries, laparoscopic surgeries create restrictions on freedom of movement due to poor ergonomic designs of surgical instruments that are long and rigid, use of pedals for controlling the operating system, fixed surgical ports for the instruments, and the location of screens [5]. All these issues would create physical fatigue and musculoskeletal disorders among surgeons. To deal with the technical challenges created by laparoscopic surgery and become proficient, laparoscopic surgeons require extensive training, expertise, and practice [6]. Advanced and structured training programs are essential to meet the surgical demands, and reduce technical limitations in laparoscopic surgeries as these MIS procedures have a steep learning curve [7]. Many new surgical methods, instruments, and devices have been developed to improve surgeons' working conditions that would help enhance the dexterity, accuracy, and ergonomics during the procedure [8]. Recently, there has been a paradigm shift from traditional open surgical procedures to more minimally invasive laparoscopic surgical techniques. This review will comprehensively explore the recent surgical techniques and technologies in laparoscopic surgeries that have emerged in order to deal with the aforementioned challenges in the field of MIS.

## Review

Methods

**Figure 1:**
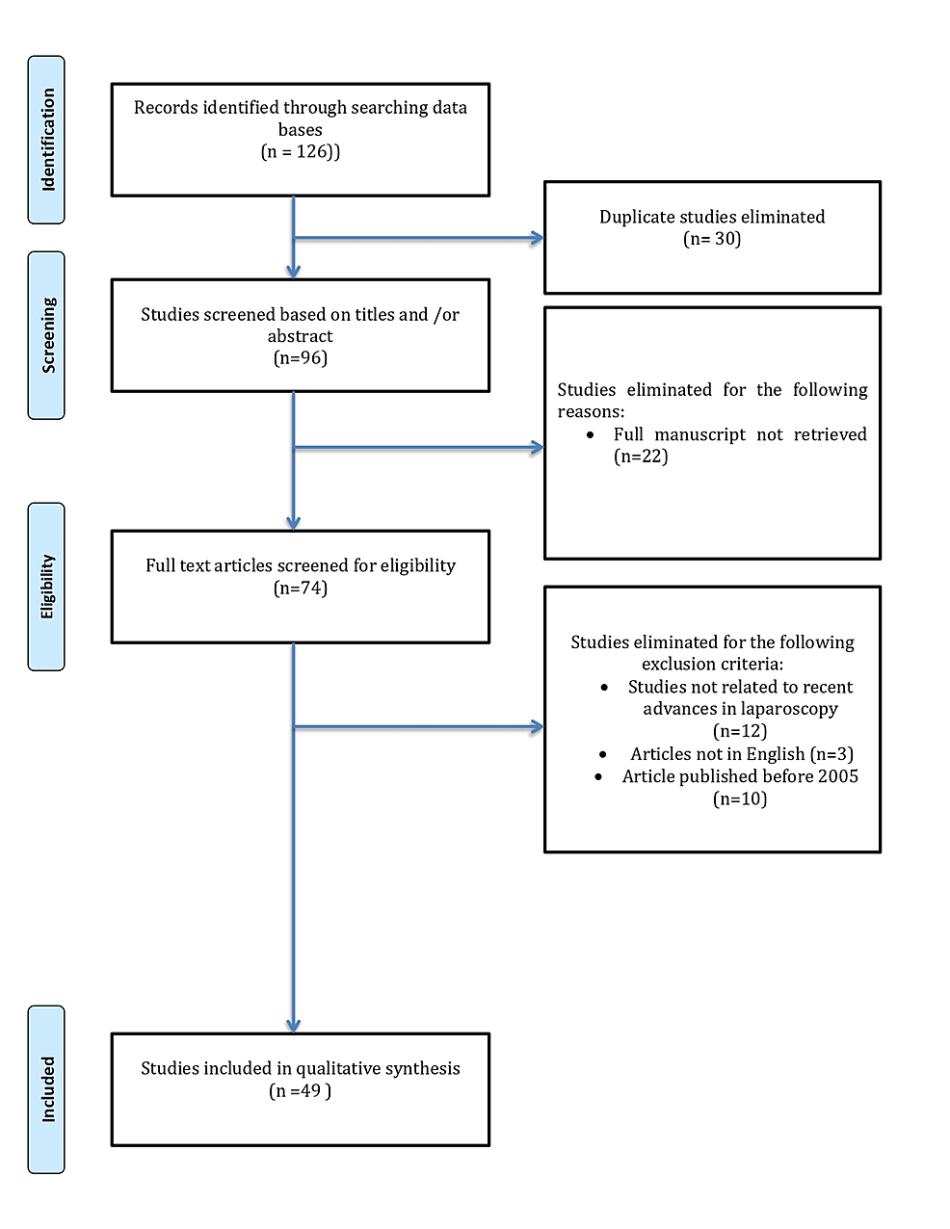
Preferred Reporting Items for Systematic Reviews and Meta-Analyses (PRISMA) flowchart for laparoscopic surgeries

A comprehensive literature search was conducted using different Internet-based search engines such as Google Scholar and bibliographic databases (PubMed, PubMed Central, MEDLINE, Medknow, EMBASE, Scopus, CINAHL, AMED) from August 2021 to October 2021. This integrative search included the following keywords and phrases: “Laparoscopic surgery OR laparoscopy AND Recent trends OR latest trends OR recent advancements”; “Laparoscopic Appendectomy AND Recent trends OR new technique”; “Minimally invasive surgery AND abdomen OR pelvis”; “Keyhole surgery AND abdomen OR pelvis.” The search was limited to articles published in the English language and those published between years 2005 and 2021. Three authors were responsible for the literature search. A total of 126 articles were initially identified. Two independent reviewers thoroughly examined the quality and content of the articles. Articles with duplicate data were excluded, and the remaining 96 articles were screened and assessed by the titles and abstracts. After a vigorous assessment, we included data from 49 articles for this review process. Bibliographic management was done using the software “EndNote” (Thomson Reuters, New York, NY) (Figure 1).

Results

Laparoscopic Appendectomy

Over the last 10-15 years, laparoscopic appendectomy (LA) has gained popularity as a result of improved diagnostic outcomes and a lower risk of wound complications. The main advantages of LA are faster recovery and return to normal activities, a better aesthetic outcome, and early resumption of oral intake compared to open surgery. With the recent development of multichannel glove ports, more complicated endoscopic operations are now possible with single-incision laparoscopic appendectomy (SILA) [9]. SILA utilizes two approaches: (a) intracorporeal laparoscopic appendectomy (ICLA) and (b) extracorporeal laparoscopic appendectomy (ECLA). In the first approach (ICLA), a 10-mm supraumbilical port is used to create pneumoperitoneum, accompanied by the placement of two 5-mm working ports well beyond the midline. Because of the parallel configuration of instruments, it requires greater technical knowledge and skills to perform the ICLA. However, many studies have shown positive outcomes with this approach [10,11]. A study that compared laparoscopy-assisted single-port appendectomy (SPA) in children with open appendectomy (OA) showed that the operative time was significantly longer in SPA than OA (60.8  min vs 57.4  min), whereas the hospital stay was found to be shorter in SPA (4.4 days vs 5.9 days) [11]. The ECLA, or video-assisted appendectomy, is a form of SILA that includes all of the initial steps of ICLA, including the establishment of pneumoperitoneum, and identification and skeletonization of the appendix. In the next phase of ECLA, the appendix is exteriorized via a 10-mm port in the right iliac fossa, and the procedure proceeds in almost the same manner as an open appendectomy. This approach typically requires two to three ports, although a number of studies have recently been reported to use the same approach using a single peri-umbilical port [12,13].

Natural Orifice Transluminal Endoscopic Surgery

Another recent development in laparoscopic surgery is natural orifice transluminal endoscopic surgery (NOTES), which utilizes access to the peritoneal cavity through natural orifices without any incisions or without passing through the anterior abdominal wall. This procedure is performed with existing endoscopic techniques using instruments in one body cavity, usually the peritoneal cavity [14]. The access to the cavity is gained via an endoscope through a natural orifice such as the mouth, anus, vagina, or urethra and/or sometimes through incisions to create internal orifices [15]. Hybrid NOTES techniques combine a NOTES method with a direct transcutaneous entry to the cavity, generally in conjunction with the laparoscopic equipment [16]. Different routes have been tried to gain entry into the peritoneal cavity that include transesophageal, transgastric, transcolonic, transvaginal, and transvesical routes.

In NOTES, the most preferred route by surgeons is stomach and a modified Seldinger dilatation or percutaneous endoscopic gastrostomy (PEG) procedure. In women, some surgeons use the vault of the vagina for access to the cavity but with certain limitations [17]. Another approach used is transrectal that uses transanal endoscopic microsurgery (TEMS) instruments. But these approaches require extreme care in both access and closure. The potential advantages of appendectomy performed with NOTES include fewer scars and reduced postoperative pain, avoidance of hernia formation, and shorter recovery time [16]. The detailed assessment of outcomes of NOTES is difficult as there are a limited number of patients treated with this approach. NOTES registries have reported that the transvesical route showed a lower complication rate (3%-8%) and technical threshold [18]. Many bariatric surgeries, such as sleeve gastrectomy (SG) or the gastrojejunostomy procedure, can now be performed using the NOTES technique. This is because NOTES procedures avoid abdominal wall incisions, while hybrid NOTES procedures minimize abdominal port sites [19]. This is certainly relevant in SG, where one of the port sites has to be made larger to remove the gastric remnant from it.

Single-Incision Laparoscopic Surgery

In the literature, various terms have been used interchangeably for this recent technique that includes single-incision laparoscopic surgery (SILS), single-port laparoscopic surgery, single-port access surgery, and transumbilical or laparoendoscopic single-site surgery [20]. Reducing the port count has shown many advantages over traditional laparoscopic surgery, including superior cosmetic outcomes, decreased discomfort and pain, faster recovery period, shorter hospital stay, and lesser port-associated complications. However, recent clinical studies in a variety of surgical specialties have failed to find substantial advantages to SILS other than cosmesis [21,22]. There is a lack of Level I and II evidence that shows benefits of SILS, and most of the reported case series show only Level IV evidence [23]. Single-incision laparoscopic splenectomy (SIL-SP) is gaining popularity even though published case reports are limited. When compared to standard laparoscopic splenectomy, SIL-SP was associated with a significantly lower conversion to open rate, lesser operative time, and similar median estimated blood loss [24]. Conventional multiport laparoscopic surgery splenectomy CMLS-SP is a procedure that is now considered the gold standard for spleen removal [25]. However, the major disadvantage of this procedure is that it requires multiple incisions compared to SIL-SP. A study done by Choi and colleagues that compared clinical outcomes of SIL-SP and CMLS-SP reported significantly no differences observed for operation time, gas passing, diet, post-operative pain, and postoperative hospital stay. However, significantly lesser blood loss was observed in CMLS-SP cases [26].

Laparoscopic Cholecystectomy

Laparoscopic cholecystectomy (LC) is a minimally invasive surgical procedure that is routinely employed for gallbladder removal nowadays. This technique is performed through four small incisions made in the abdomen for insertion of long surgical instruments and a surgical video camera [27]. This procedure has replaced open cholecystectomy for various conditions such as cholecystitis (acute/chronic), acalculous and symptomatic cholecystitis, cholelithiasis, biliary dyskinesia, gallstone pancreatitis, and gallbladder masses/polyps. Common bile duct (CBD) stones are one of the conditions where general surgeons commonly use LC. Although advanced laparoscopic surgical skills are necessary to conduct the operation, single-stage laparoscopic CBD exploration (LCBDE) during LC is currently predominantly utilized to treat cholecystocholedocholithiasis [28]. However, it was reported by Connor and Garden that the incidence of bile duct injury is slightly higher in LC compared to the open technique [29]. Despite this, LC is the preferred procedure for CBD stones because it provides lesser postoperative discomfort and pain, a shorter hospital stay, improved cosmetics, and higher patient satisfaction [30]. In addition, an intraoperative cholangiography could be used to prevent CBD injury, although the injury infrequently occurs during LC [31]. A recent retrospective analysis of gallbladder removal surgeries in Saudi Arabia reported that LC was the commonest mode of surgical removal (91.5%); open surgery was used in only 5.8% cases whereas 2.7% cases were converted from LC to open technique during the surgery [32].

Laparoscopic Bariatric Surgery

This surgical procedure has gained wide popularity among surgeons and is the commonly performed laparoscopic surgery after LC. Many bariatric procedures such as intestinal bypass, vertical banded gastroplasty (VBG), and laparoscopic adjustable gastric banding (LAGB) have been used to manage obesity. However, many of these procedures are abandoned as a result of the emergence of novel methods of bariatric surgery [33]. For the last six years, laparoscopic sleeve gastrectomy (LSG) has become the common bariatric surgical procedure performed for weight loss in the majority of the countries. Some of the other newer laparoscopic procedures include laparoscopic Roux-en-Y gastric bypass (LRYGBP), one anastomosis gastric bypass (OAGB)/mini-gastric bypass (MGB), and LAGB [34-36]. LSG is a completely restrictive bariatric procedure and has been suggested as a potential first-stage surgery before more complex techniques such as biliopancreatic diversion (BPD) with duodenal switch or LRYGBP. The surgery involves removing 75%-80% of the stomach, leaving only a stomach sleeve. This section of the stomach limits the quantity of food a patient may eat at first, resulting in considerable weight loss. LRYGBP is one of the commonly performed procedures after LSG. In this procedure, the food intake is restricted by creating a small gastric pouch, which paves the way to bypass a big portion of the small intestine [33]. Although there are no significant differences observed in weight loss between LSG and LRYGBP after three months, the weight loss is comparatively more significant in LSG after six and nine months [37,38]. A recent report from the Middle East region showed that LSG was the commonest procedure performed for weight loss/obesity, followed by LRYGB, OAGB/MGB, and LAGB [39].

Laparoscopic Anti-Reflux Surgery

Recent advancements in laparoscopic fundoplication methods have rekindled interest in the surgical management of gastroesophageal reflux disease (GERD), resulting in the reporting of considerable clinical series [40]. Laparoscopic inguinal hernia repair and laparoscopic colonic resection have been questioned for the possibility of causing new and different complications than those observed with open procedures [41]. Various laparoscopic anti-reflux surgery techniques are available today that include Dor fundoplication, an anterior 180-degree wrap; Toupe fundoplication, a posterior 270-degree wrap; and Nissen fundoplication, a total posterior 360-degree wrap. Laparoscopic Nissen fundoplication (LNF) has become the gold standard in anti-reflux surgery for chronic and unmanageable GERD. Studies show contrasting findings between partial fundoplication (PF) and LNF, where two meta-analyses reported fewer reoperations and better functional outcomes with PF [42,43]. In contrast, some retrospective studies favored the LNF for its superior reflux control [44,45]. Clinical results after LNF seem to be comparable irrespective of whether the short gastric vessels are separated. With the advent of new energy sources, separating the short gastric vessels is still advised to mobilize the fundus and minimize fundus stress. However, about 2.85%-4.4% cases undergoing LNF show recurrence, and the majority of them need revision surgery [46].

Laparoscopic Cancer Resection

Laparoscopic gastrectomy is considered the gold standard for patients with early-stage stomach cancer or those who need palliative care. The advantages of this procedure include shorter hospital stays, lesser postoperative pain, and improved quality of life after the procedure [47]. Laparoscopy-assisted distal gastrectomy with extracorporeal anastomosis is commonly done in many developed countries [48]. Many clinical trials have shown the short-term advantages of laparoscopic colorectal cancer resection and acceptable oncological outcomes with lower recurrence rates [49,50]. Recently, techniques like NOTES and single-incision laparoscopic surgery (SILS) have shown promising clinical outcomes for colorectal cancer resection [51,52]. A study done in Saudi Arabia that compared survival rates between laparoscopic cancer resection (LCR) and open curative resection for potentially curable colon cancer demonstrated that LCR showed significantly a higher survival rate than open approach (90.3% vs 76.7%) [53].

Laparoscopic Pancreatic Surgery

Recent technological advancements in surgical methods have made it possible to do laparoscopic surgery for various pancreatic conditions [54]. The technique of laparoscopic distal pancreatic resection, originally described by Gagner in 1996, has acquired widespread popularity due to its procedural simplicity and the avoidance of anastomosis [55].

Laparoscopic Hepatic Surgery

The oncological outcomes of laparoscopic liver resection have been found to be superior to open surgery with lesser postoperative complications. However, laparoscopic hepatic surgery must be proven as a safe and effective alternative to open liver resection and for the treatment of hepatocellular cancer [56]. Additional research is needed to determine the oncologic and patient-centered results of these novel technologies. A recent meta-analysis showed that a combination of laparoscopic surgery and radiofrequency ablation is superior to resection alone for treating colorectal liver metastases [57].

Robot-Assisted Laparoscopic surgery

Robot-assisted laparoscopic surgery (RALS) has opened new avenues and has overcome the drawbacks of conventional LS, giving better ergonomics and enhanced dexterity and orientation, availability of numerous instrumental tips for the EndoWrist® instrument (Intuitive Surgical, Sunnyvale, CA), three-dimensional visualization, and tremor reduction [58]. RALS prostatectomy and RALS hysterectomy were the two common procedures done during the last decade. But, recently, RALS has been employed for many surgeries such as nephrectomy (simple and radial), adrenalectomy, pyeloplasty, ureteroureterostomy, and bladder diverticulectomy with ureteric re-implantation [59]. A recent meta-analysis reported that RALS has no major advantages over LS and open surgeries. In addition, RALS has reduced flexibility, requires increased operative time, and it is expensive compared to other LS procedures [60]. However, RALS has a shorter learning curve compared to LS and open surgeries. A study done among urologists in Saudi Arabia reported that 40% of them used surgical robots for laparoscopic surgeries and 23.2% reported that they have obtained fellowship in RALS [61].

## Conclusions

LS is considered an effective technique in almost every abdominal surgery procedure and is recommended, as evident by numerous scholarly publications in this field. Level I evidence demonstrating the advantage of LS over open surgery has been reported for numerous operations, including fundoplication for GERD, bariatric surgery for weight loss, and cancer resection. Advanced LS has subsequently been expanded to include hepatectomy, pancreatectomy, urology, and gynecology. However, patients who are at risk of having elevated abdominal pressure during LS should proceed with care. Recent advances in NOTES, SILS, and RALS are promising, although robotic laparoscopy has increased operative timing and needs extensive training. Further studies are needed.
